# Direct-from-blood RNA sequencing identifies the cause of post-bronchoscopy fever

**DOI:** 10.1186/s12879-019-4462-9

**Published:** 2019-10-28

**Authors:** Emily R. Ko, Casandra W. Philipson, Thomas W. Burke, Regina Z. Cer, Kimberly A. Bishop-Lilly, Logan J. Voegtly, Ephraim L. Tsalik, Christopher W. Woods, Danielle V. Clark, Kevin L. Schully

**Affiliations:** 1 Center for Applied Genomics and Precision Medicine, Duke University School of Medicine, Durham, NC 27708 USA; 2 Department of Hospital Medicine, Duke Regional Hospital, Durham, NC 27705 USA; 3 Genomics and Bioinformatics Department, Biological Defense Research Directorate, Naval Medical Research Center-Frederick, Fort Detrick, Frederick, MD USA; 4 Defense Threat Reduction Agency, Fort Belvoir, VA USA; 5 Leidos, Reston, VA USA; 6 Division of Infectious Diseases, Duke University School of Medicine, Durham, NC 27710 USA; 7 Emergency Medicine Service, Durham VA Health Care System, Durham, NC 27705 USA; 8 Medicine Service, Durham VA Health Care System, Durham, NC 27705 USA; 9 Austere environments Consortium for Enhanced Sepsis Outcomes (ACESO), Biological Defense Research Directorate, Naval Medical Research Center-Frederick, 8400 Research Plaza, Fort Detrick, MD 21702 USA

**Keywords:** Transcriptome, Metagenomic sequencing, Early diagnosis, Molecular diagnostics, Bacterial infections, *Stenotrophomonas maltophilia*

## Abstract

**Background:**

Antibiotic resistance is rising at disturbing rates and contributes to the deaths of millions of people yearly. Antibiotic resistant infections disproportionately affect those with immunocompromising conditions, chronic colonization, and frequent antibiotic use such as transplant patients or those with cystic fibrosis. However, clinicians lack the diagnostic tools to confidently diagnose and treat infections, leading to widespread use of empiric broad spectrum antimicrobials, often for prolonged duration.

**Case presentation:**

A 22 year-old Caucasian female with cystic fibrosis received a bilateral orthotopic lung transplantation 5 months prior to the index hospitalization. She underwent routine surveillance bronchoscopy and was admitted for post-procedure fever. A clear cause of infection was not identified by routine methods. Imaging and bronchoscopic lung biopsy did not identify an infectious agent or rejection. She was treated with a prolonged course of antimicrobials targeting known colonizing organisms from prior bronchoalveolar lavage cultures (Pseudomonas, *Staphylococcus aureus*, and *Aspergillus*). However, we identified *Stenotrophomonas maltophilia* in two independent whole blood samples using direct-pathogen sequencing, which was not identified by other methods.

**Conclusions:**

This case represents a common clinical conundrum: identification of infection in a high-risk, complex patient. Here, direct-pathogen sequencing identified a pathogen that would not otherwise have been identified by common techniques. Had results been clinically available, treatment could have been customized, avoiding a prolonged course of broad spectrum antimicrobials that would only exacerbate resistance. Direct-pathogen sequencing is poised to fill a diagnostic gap for pathogen identification, allowing early identification and customization of treatment in a culture-independent, pathogen-agnostic manner.

## Background

The last two decades have seen a rapid rise in antibiotic-resistant infections, triggering alarm within global communities [1]. Nearly 10 million additional annual deaths due to antimicrobial resistance are predicted to occur by 2050 at a cost of $100 trillion [2]. Despite antibiotic stewardship programs and increased awareness, clinicians continue to prescribe antibiotics without clear evidence of bacterial infection, especially in high risk populations such as immunocompromised patients, children, and the elderly. It is well recognized that early and appropriate antibiotics reduce septic shock mortality [3]. Although similar data about the timing of antibiotics in less acutely ill patients is lacking, it remains common clinical practice to prescribe broad, empiric antibiotic therapy as a precaution. One of the greatest impediments to more appropriate antibiotic utilization is the lack of diagnostic tools to identify when antibiotics can be safely withheld, delayed, or stopped. Standard clinical diagnostics for detection of bacterial pathogens rely heavily on culture-based methods, which require incubation and relatively large blood volumes for detection. Furthermore, these techniques may not reliably detect some slow growing or fastidious organisms. Whereas antibiotic stewardship is essential to slowing the development and spread of antibiotic resistance, these programs are most effective when coupled with improved diagnostics for infectious disease [4].

Metagenomic sequencing has significant potential as a complimentary routine molecular diagnostic strategy for infectious diseases. Advantages of metagenomic sequencing include its unbiased nature, detection of unculturable organisms, and the potential to obtain strain typing information as well as genotypic susceptibility. Despite these advantages, there are challenges to overcome. Sensitivity is lower for organisms with small genomes or pathogens present at low titers. Also, contaminating sequences can be introduced from the environment, laboratory reagents [5] (Naccache, Hackett, Delwart, & Chiu, 2014), run-to-run carryover, or from bleed-through among multiplexed samples or index cross-contamination [6] (P. E [7].). The resulting massive, complex data require specialized tools and expertise for analysis. Interpreting results is not yet standard in clinical labs or in clinical practice. There are technical challenges and financial considerations as well. Current methods typically require 24 h or more to generate a result. Although this is faster than culture in many cases, it is too long to inform initial treatment decisions. Furthermore, the cost is substantial when compared to existing microbiological diagnostic tools. However, sequencing does not replace existing clinical microbiology costs and thus, in most cases, it represents an added expense. There are, of course, anecdotes where sequencing could have saved time, money, and most importantly lives. However, delineating when to perform this type of test and in which patients has yet to be defined. Notwithstanding these challenges, there are many examples of metagenomic sequencing approaches providing new insights into the etiology of infections (M. R [8].). Perhaps as a starting point, this new diagnostic strategy is best considered in patients at highest risk and for whom conventional microbiological testing is challenging. In this report, we describe the application of such technology to an immunocompromised patient with cystic fibrosis (CF) and lung transplant.

Fiberoptic bronchoscopy (FOB) and bronchoalveolar lavage (BAL) are routinely used to monitor and treat patients with chronic lung disease or lung transplant. Post-bronchoscopy fever develops up to 37% of the time in children and 16% of the time in adults [9–15]. Fever is more common in patients with focal bronchitis, known infections, and age less than two or over sixty [9, 11, 14, 16]. Case studies have reported transient bacteremia [10, 11, 17] and cytokine release [18, 19] after bronchoscopy, offering an explanation for the fever. However, blood culture data is insensitive and transient bacteremia is rarely reported [10, 11, 17]. Cytokine release is nonspecific and could be related to inflammation from the procedure, transient infection, or the disease process that led to the bronchoscopy in the first place.

We present the case of a 22 year-old female with CF five months post bilateral orthotopic lung transplantation who had a routine surveillance bronchoscopy performed. That procedure was complicated by fever and systemic inflammatory response. We retrospectively identified the cause as *Stenotrophomonas maltophilia* bacteremia using direct-from-blood RNA sequencing. This case report highlights the application of a metagenomic sequencing technology to a poorly characterized condition and how this information could impact clinical decision making.

The patient was enrolled at Duke University Hospital as part of the Austere environments Consortium for Enhanced Sepsis Outcomes (ACESO) Study to identify early host-based determinants of sepsis. This study was a multi-center clinical trial conducted at Duke University Medical Center and hospitals in Cambodia, Ghana, Liberia, and Uganda in which patients who met two of four systemic inflammatory response syndrome (SIRS) criteria [20] were enrolled. Studies were approved by relevant Institutional Review Boards (IRBs) and in accordance with the Declaration of Helsinki. After providing written informed consent, blood samples were collected in PAXgene Blood RNA tubes (BD Biosciences) and nasopharyngeal swabs were collected for respiratory pathogen testing. All other laboratory analysis and culture results were obtained through routine clinical care and obtained from the medical record.

## Case presentation

A 22 year-old female with end stage lung disease secondary to CF underwent bilateral orthotopic lung transplant (BOLT) five months prior to enrollment in our study. The patient’s pre-transplant history was notable for airway colonization with mucoid Pseudomonas and *Stenotrophomonas maltophilia*, Methicillin Resistant *Staphylococcus aureus* (MRSA), and Aspergillus in addition to severe chronic sinusitis. Her post-transplant history was remarkable for multidrug resistant pseudomonal infection of her surgical incision, *C. tropicalis* bloodstream infection, and mild acute cellular rejection (ACR stage A1Bx) although none of these were active issues at the time of presentation.

Six weeks prior to presentation to the emergency department (ED), the patient began having persistent low-grade fevers of 99-101 °F. She was treated for a possible urinary tract infection with a course of ciprofloxacin due to an abnormal urinalysis but urine culture only grew mixed flora without a predominant pathogen. She continued to have low grade fevers and was treated with a course of levofloxacin for nonspecific pulmonary complaints but without clear evidence of infection on chest CT. The patient returned to clinic two weeks prior to enrollment with continued low grade fevers and was started on tobramycin nasal washes for mild sinus symptoms. The trimethoprim/sulfamethoxazole she used for *Pneumocystis jirovecii* prophylaxis was changed to pentamidine due to concerns about drug-induced fever. She was scheduled for outpatient bronchoscopy to monitor for infection and rejection as a possible cause of her persistent fevers.

At the time of bronchoscopy, her fevers had completely resolved and she reported feeling well without new symptoms. The patient underwent the scheduled bronchoscopy with bronchoalveolar lavage (BAL) and biopsies. Approximately 12 h later, the patient began having fevers and chills at home, which led her to come to the ED. She demonstrated a temperature of 103.1 °F, heart rate of 124 beats/minute, white blood cell count of 13.8 × 10^9^ cells/uL (Ref 3.2–9.8 × 10^9^ cells/uL) and lactate of 4.2 mmol/L (Ref 0.5–2.2 mmol/L). All other vital signs and laboratory analysis were within normal limits (Table 1). Two sets of blood cultures and urine culture showed no growth. Cytomegalovirus (CMV) and Epstein Barr Virus (EBV) quantitative PCR testing were negative. Cultures from the bronchoscopy performed one day prior to presentation grew rare mucoid Pseudomonas, rare MRSA, and Aspergillus. Respiratory viral pathogen PCR panel did not demonstrate viral pathogens on either routine clinical testing or supplemental study testing. Pathology did not show evidence of acute cellular rejection or infection. The patient was started on broad spectrum antibiotics with vancomycin and piperacillin/tazobactam at the time of enrollment. All of her presenting signs and symptoms, vital signs and laboratory testing were within normal limits within 24 h of admission. Biopsy from BAL and chest imaging did not reveal evidence of invasive infection. CT chest revealed ground glass opacities in the region where the BAL and biopsies were performed, interpreted by treating clinicians to be procedure related. The clinical team discharged the patient with two weeks of IV doxycycline, three weeks of IV piperacillin/tazobactam, and three months of voriconazole to cover bacterial and fungal elements noted on BAL, although no clear sources of infection were discovered.

**Table 1:** Clinical information, Laboratory Procedures and Results

Demographics		22 year old Caucasian female
Symptoms		fevers, chills, nausea, poor appetite
Vital signs	Blood pressure:	107/57
	Heart Rate:	124
	Respiratory rate:	22
	Oxygen saturation:	96% room air
Physical Exam	Lung:	mild wheeze and scattered crackles
	otherwise, unremarkable	
Laboratory analysis	WBC:	13.2
	Hemoglobin:	8.5
	Platelets:	279
	Sodium:	137
	Potassium:	4.8
	BUN:	14
	Creatinine:	1.2
	Glucose:	289
	Lactate:	4.2
	Thyroid Panel*:	Within normal limits
	ESR & CRP:	within normal limits
	ANA Screen*:	Negative
Microbiology	Blood Culture:	no growth
	Urine Culture:	no growth
	BAL Culture:	rare Mucoid Pseudomonas
		rare MRSA
		*Aspergillus flavus* present
	CMV quantitative PCR*:	undetectable
	EBV quantitative PCR*:	undetectable
Pathology	BAL biopsy:	no evidence of rejection or infection
Radiology	Chest X-ray:	right lower lobe atelectasis
	CT scan chest:	Interval development of ground glass and dense airspace
		opacities in the lateral basal segment of the right lower
		lobe. Recent bronchoscopy with biopsy in this location.
Nasopharyngeal Swab	Collected using the BD Universal Transport System from BD Biosciences using routine procedures and analyzed by multiplex PCR (xTAG® Respiratory Viral Panel FAST v2)	
RNA Sequencing	Total RNA was isolated from 2.5 mL of peripheral blood drawn directly into PAXgene Blood RNA stabilization tubes (BD Biosciences) using the PAXgene Blood miRNA Kit (Qiagen). Abundant Globin and ribosomal RNA transcripts were depleted using Globin-Zero Gold rRNA Removal Kit (Illumina, San Diego, CA, USA). RNA sequencing library preparation used NEBNext Ultra RNA Library Prep Kit for Illumina (NEB, Ipswich, MA, USA). The resulting library was sequenced on the Illumina HiSeq 4000 to produce at least 50 million reads in a 2X 150 bp paired end format. Sequences were deposited in the Sequence Read Archive under project PRJNA408161.	

*indicates test was done within 1 month of enrollment. All other tests were done at the time of enrollment or are the results of the BAL done the prior day

WBC = white blood count, BUN = blood urea nitrogen, ESR = erythrocyte sedimentation rate, CRP = c-reactive protein, ANA = anti-nuclear antibody, BAL = bronchoalveolar lavage, CMV = Cytomegalovirus, EBV = Epstein Barr virus

At the time of her initial evaluation in the ED, the patient was identified for enrollment in a research study on host-based biomarkers for sepsis: The Austere environments Consortium for Enhanced Sepsis Outcomes (ACESO). As part of study-related activities, we obtained a blood sample for RNA analysis.

**Fig. 1:**
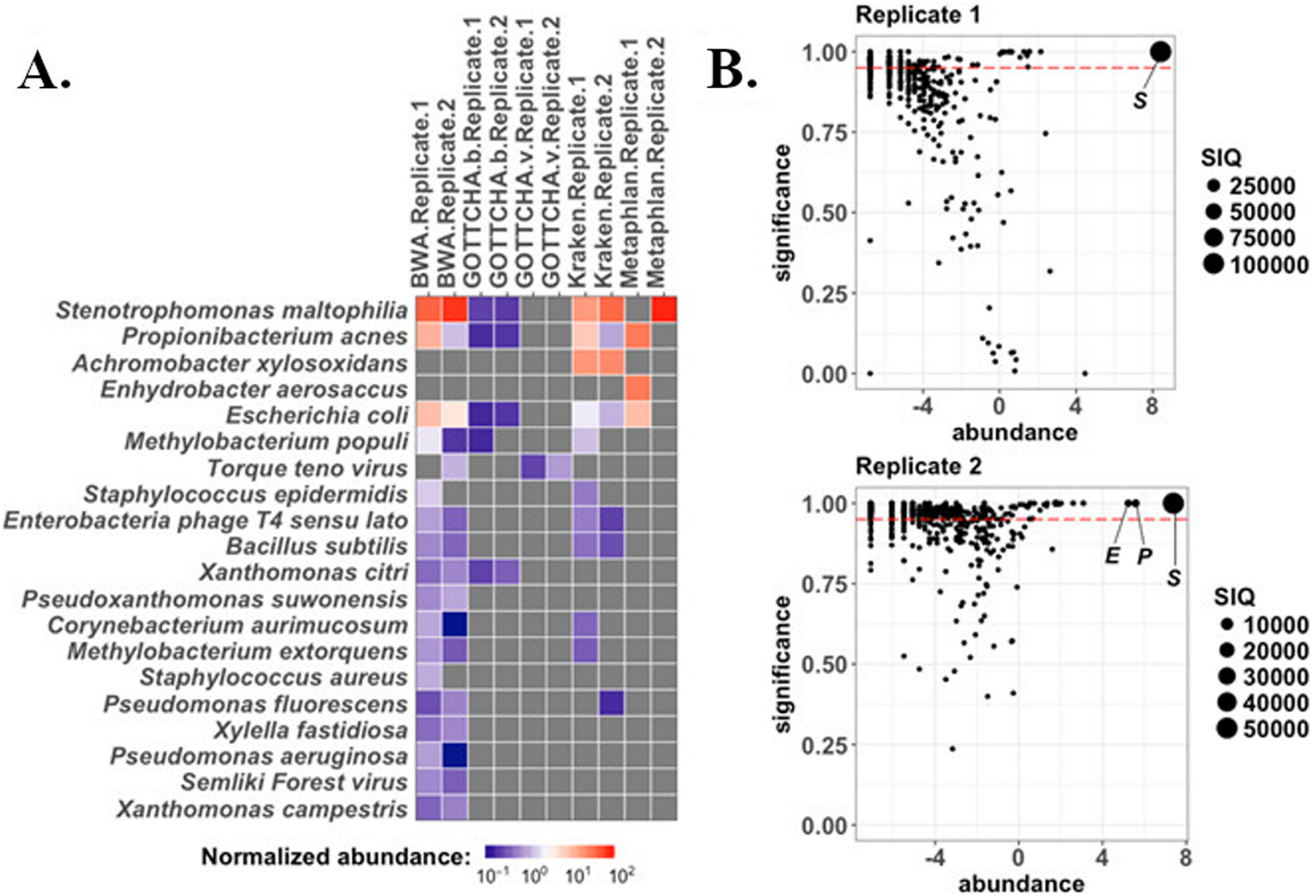
High-throughput sequencing based microbial detection in replicate samples from blood. **a** Quality controlled reads from two replicates were profiled by four different taxonomic classifiers: BWA-mem against RefSeq; GOTTCHA against a bacterial database (GOTTCHA.b); GOTTCHA against a viral database (GOTTCHA.v); Kraken-mini; and MetaPhlAn. Normalized read counts mapping to the top 20 organisms by one or more tools are presented at the species level (heatmap). **b** The SIQ score was calculated for each replicate using reads that mapped to RefSeq using BWA-mem. Coordinates on SIQ plots are relative abundance of all species (*x-axis*), by significance (1 – *p* value, *y-axis*), and the size of the dot represents the SIQ score. The dashed red line indicates a *p* value of 0.05. Microbes detected at significant levels with a normalized abundance level > 4 are labeled: *S*, *Stenotrophomonas maltophilia; P, Propionibacterium acnes; E, Escherichia coli.* Plots were generated using R statistical programming

Metagenomic sequencing was performed on RNA isolated from peripheral blood collected at the time of this visit, which was five months after lung transplant. Blood samples were stabilized in two independent PAXgene Blood RNA tubes yielding Replicates 1 and 2, respectively; replicates were sequenced on different sequencing runs. EDGE Bioinformatics Software version 1.5 (P. E [7].) was used to classify the composition of microbes in the subject’s peripheral blood sample. All EDGE parameters for taxonomy tools were set to default as described (Philipson, 2017). Reads that were classified by BWA-mem mapping to RefSeq were used to calculate a sepsis indicating quantifier (SIQ) score as previously described [21]. All non-human reads were assigned a taxonomic classification by three separate algorithms and five separate databases (Fig. 1A). *Stenotrophomonas maltophilia* was the dominant microbe detected by all tools screening against bacterial databases for Replicate 2. Results were reproduced in Replicate 1 except the MetaPh1An algorithm did not detect the pathogen in Replicate 1, which was due to different sequencing depths for the independent runs. When detected, the top hit for all tools agreed at the strain level for *S. maltophilia* strain D457. The SIQ score [21] was calculated using classification results from the BWA-mem algorithm to ascertain the significance of each microbe as an etiological agent of bacteremia versus a false positive result. *S. maltophilia* taxonomy assignments resulted in the highest SIQ score for both replicates (Fig. 1B). Additionally, the pattern of SIQ scores indicates *S. maltophilia* was the singular dominant microbe in the patient’s blood samples.

## Discussion

We report a case of post-bronchoscopy fever in a patient with CF and lung transplantation. Clinical laboratory analysis, imaging, and culture results could not confirm the presence of an active infection but could not exclude it either. Uncertainty about the presence of active infection in the setting of colonization led the clinical team to treat this patient with a prolonged course of broad spectrum antimicrobials. This patient represents a population at high risk for the acquisition of antibiotic resistant infections, significantly increasing the risk of morbidity and mortality from both the infection and the antibiotics used to treat it [22]. However, delaying antibiotics or undertreating a bacterial infection in an immunocompromised patient could have dire consequences. Clinicians commonly face the need to limit antibiotic use to avoid antibiotic resistance, but they lack sufficiently sensitive diagnostics to exclude the presence of an active bacterial infection. Our results demonstrate that use of direct pathogen sequencing can augment routine testing for bacterial pathogens and offer timely solutions to direct treatment and guide more appropriate antimicrobial use.

Etiologies of infection in CF patients are broad and often complicated due to chronic colonization of multiple opportunistic microbes [22]. Identification of organisms, especially from non-sterile sites such as sputum, may simply represent commensal colonization rather than infection. As demonstrated here and by others, unbiased metagenomic sequencing can be used to characterize complex disease landscapes, especially for diagnostically challenging pulmonary diseases in which multiple organisms contribute to a pathogenic microbiome [23, 24]. In addition to being unbiased, direct sequencing from blood is less invasive than routine bronchoscopy, mitigating associated procedural risks. Importantly, detection of bacterial agents from a nonsterile source, such as sputum or bronchoalveolar lavage, does not necessarily indicate infection and could be representative of the microbiota. However, direct sequencing of pathogenic bacteria from blood, which is expected to be sterile, is more indicative of infection. Furthermore, metagenomic sequencing has demonstrated utility for diagnosing infections, yielding more sensitive and specific results than standard of care methods [25].

This case report demonstrates that multi-tool bioinformatics analysis followed by statistical mathematical normalization can bolster confidence for diagnostic determination and minimizing false positive results. Combining results from multiple tools has been demonstrated to increase the likelihood of detecting true positives in metagenomic datasets because the overlap in detection between sets of tools almost always increases precision when compared to using tools individually in benchmarking studies [26]. Two replicates were sequenced and processed by four separate taxonomic classifiers that vary in sensitivity and specificity. Results demonstrate that three of four detection methods agreed; MetaPhlAn did not detect *S. maltophilia* in Replicate 1. MetaPhlAn is one of the most specific community profiling tools, and detection in one sample only is likely a result of a difference in the depth of sequencing achieved on the independent runs. Given the output from all other tools, this result demonstrates the utility of replicates and a multi-tool approach for metagenomics-aided diagnostics.

A common problem faced by clinicians caring for CF patients is distinguishing colonization from infection. In this patient, rare growth of *Pseudomonas aeruginosa*, *Staphylococcus aureus,* and *Aspergillus* in bronchoalveolar lavage samples (Table 1) was observed. While both *S. aureus* and *P. aeruginosa* were detected at low levels in blood samples by BWA, neither bacterial species were detected in quantities deemed significant by SIQ analysis, further arguing against an active *P. aeruginosa* or *S. aureus* infection. Sequences corresponding to *Propionibacterium acnes* and *Escherichia coli* were also prevalent at significant levels in one replicate. While *P. acnes* is considered an opportunistic pathogen, *Propionibacterium* species found in blood cultures are often determined to be contaminants especially when found in only a single replicate ([27, 28],(J. R [29].). Furthermore, *E. coli* DNA is known to contaminate sequencing reagents and is frequently detected in sequence-based studies [5]. Thus, *S. maltophilia* was confidently identified as the singular dominant microbe in the patient’s blood samples. Moreover, detection as the dominate microbe through analysis of RNA sequencing (e.g. replicating bacterial transcripts) by multiple algorithms suggests an active infection in the patient’s blood.

*S. maltophilia* is a rare but serious cause of infection and is associated with a 21–69% fatality rate in immunocompromised individuals with bacteremia [30–33]. It is a gram-negative pathogen that evades immune clearance due to its intracellular localization. It typically forms biofilms on tubing and on the respiratory epithelium of dysfunctional airways. Interestingly, *S. maltophilia* did not grow in BAL cultures at the time of enrollment but was detected in two independent blood samples by direct-pathogen sequencing. This can be explained, in part, by the fact that *S. maltophilia* has a relatively slow growth rate and can be missed if growth plates are not carefully scrutinized. The presence of *S. maltophilia* in BAL cultures prior to and after enrollment indicated this bacteria was part of this patient’s complex lung microbiota. Invasive procedures, such as BAL with biopsies, as in this case, can increase the risk for bacterial translocation into the blood stream [34–36], and detection from two independent blood samples reveals that translocation of *S. maltophilia* into the bloodstream occurred in this case. Importantly, the presence of bacteremia was undetected with clinically available cultures. Clinical knowledge of these results could have allowed pathogen directed antimicrobial therapy instead of a prolonged course of broad spectrum antibacterial and antifungal agents. This knowledge is more critical in the age of antibiotic resistance, where clear detection of a pathogen could facilitate appropriate treatment early in the clinical course avoiding morbidity and mortality. For example, *S. maltophilia* has a β-metallocarbapenemase that makes it constitutively resistant to carbapenems, an antibiotic class commonly employed in this patient population. Fortunately, the broad spectrum antibiotics used in this case were effective against *S. maltophilia*, although they were not the treatment of choice.

This case highlights the importance of developing improved diagnostics for identification of the presence of infection and delineation of the pathogens involved. Patients are often exposed to over treatment with broad spectrum antimicrobials, even when the presence of infection is unclear. More importantly, the choice of treatment may be incorrect as infections are caused by increasingly diverse and resistant pathogens. Metagenomic sequencing-based diagnostics complement existing pathogen detection techniques, but may provide a new agnostic method that is not prone to the pitfalls of culture. Another advantage to metagenomics technologies is the ability to detect emerging pathogens due to its unbiased nature. The cost and turnaround time for metagenomic diagnostics are continuously improving, making extension to clinical use an increasingly viable option. Clinical laboratories have published case reports presenting sample-to-answer metagenomic sequencing protocols that can be performed routinely in a 30–48 h time frame [8, 21, 37]. Despite its advantages, interpreting raw shotgun sequencing data for infectious diseases remains challenging. Two primary risks of sequence-based diagnostics are false positives and false negatives resulting in inappropriate or inadequate treatment, respectively. One method to circumvent the aforementioned risk is to confirm agreement among several bioinformatics tools that screen sequences against comprehensive genomic databases with varying degrees of sensitivity and specificity. Mathematical approaches such as the SIQ score [21] and weighted z-score [38] have also been developed to simplify and prioritize microbes identified by bioinformatics algorithms.

In conclusion, rapid pathogen sequencing techniques are poised to fill the gap in pathogen identification, allowing early identification and customization of treatment once the costs, speed, and availability of sequencing further improves. This strategy is not unique to bacterial pathogen identification but applies to any pathogen present in a particular sample including viruses, fungi, or parasites.

## Data Availability

The datasets used and/or analyzed during the current study are available from the corresponding author on reasonable request.

## **Abbreviations**

ACESO: Austere environments Consortium for Enhanced Sepsis Outcomes
ACR stage: Acute cellular rejection
ANA: Anti-nuclear antibody
BAL: Bronchoalveolar lavage
BOLT: Bilateral orthotopic lung transplant
BUN: Blood urea nitrogen
CF: Cystic fibrosis
CMV: Cytomegalovirus
CRP: c-reactive protein
EBV: Epstein Barr virus
ED: Emergency department
ESR: Erythrocyte sedimentation rate
FOB: Fiberoptic bronchoscopy
L: Liter
mmol: millimol
MRSA: Methicillin Resistant *Staphylococcus aureus*
SIQ: Sepsis indicating quantifier
SIRS: Systemic inflammatory response syndrome
uL: Microliter
WBC: White blood count
